# Modeling out-of-plane actuation in thin-film nematic polymer networks: From chiral ribbons to auto-origami boxes via twist and topology

**DOI:** 10.1038/srep45370

**Published:** 2017-03-28

**Authors:** Vianney Gimenez-Pinto, Fangfu Ye, Badel Mbanga, Jonathan V. Selinger, Robin L. B. Selinger

**Affiliations:** 1 Beijing National Laboratory for Condensed Matter Physics and CAS Key Laboratory of Soft Matter Physics, Institute of Physics, Chinese Academy of Sciences, Beijing, China; 2 Kent State University, Liquid Crystal Institute, Kent OH, United States; 3 Department of Chemical Engineering, Columbia University, New York, NY, United States; 4 School of Physical Sciences, University of Chinese Academy of Sciences, Beijing, China; 5 Chemical and Petroleum Engineering Department, University of Pittsburgh, Pittsburgh, PA United States

## Abstract

Various experimental and theoretical studies demonstrate that complex stimulus-responsive out-of-plane distortions such as twist of different chirality, emergence of cones, simple and anticlastic bending can be engineered and pre-programmed in a liquid crystalline rubbery material given a well-controlled director microstructure. Via 3-d finite element simulation studies, we demonstrate director-encoded chiral shape actuation in thin-film nematic polymer networks under external stimulus. Furthermore, we design two complex director fields with twisted nematic domains and nematic disclinations that encode a pattern of folds for an auto-origami box. This actuator will be flat at a reference nematic state and form four well-controlled bend distortions as orientational order changes. Device fabrication is applicable via current experimental techniques. These results are in qualitative agreement with theoretical predictions, provide insight into experimental observations, and demonstrate the value of finite element methods at the continuum level for designing and engineering liquid crystal polymeric devices.

Liquid Crystal Elastomers combine the orientational order of liquid crystals and the elastic properties of rubber. They are formed by linking liquid crystal mesogens with chains in a polymer network^1^, which brings controllable anisotropy in an otherwise isotropic polymeric material. Coupling between the mesogens orientational order and the polymer backbone produces macroscopic shape deformations that can be driven by temperature change, changes in chemical environment^2^, applied electric field^3^, or illumination^4^,^5^,^6^,^7^. For the case of a LCE cross-linked in the nematic phase, the sample’s director microstructure controls its shape evolution under temperature change. LCE with a uniform nematic director undergo a homogeneous contraction/elongation of the sample when temperature *T* changes^3^. Other 3-D shape distortions such as twisting, bending, anticlastic (saddle-like) bending and formation of cones can be engineered by “blueprinting” complex director patterns into the LCE at the time of cross-linking^8^,^9^.

Previous studies by Urayama and coworkers^10^ studied thermal-responsive actuation of LCE films with a 90° twist in director orientation along the sample thickness. These twisted nematic geometries can have on-axis mid-plane director^10^ - either along the sample short axis (S-geometry) or long axis (L-geometry) – or off-axis mid-plane director given by an offset angle. Experiments and theory revealed these ribbons form curly helicoid/spiral morphologies and switch helical sense with temperature^10^. Recently, Katsonis and coworkers^11^,^12^ synthetized biomimetic photo-responsive elastic springs whose actuation is encoded by a 90-degree-twist liquid crystalline microstructure with different angular orientation. They observe a variety of morphologies given liquid crystal polymer composition and asymmetry sources such as: twist handedness, angular offset of the mid-plane director and gradient in crosslinking density along the sample thickness. Finite element simulations at the continuum level are valuable for analysis and prediction on shape evolution of rubbery liquid crystalline polymer solids given their complex “blueprinted” microstructure. Numerical studies have explained the temperature-induced morphing on narrow off-axis TNE ribbons. Here, in the light of analytical predictions^10^ and experimental realizations^11^, we numerically demonstrate different arising morphologies in on-axis TNE ribbons as a function of sample aspect ratio and changes in nematic order.

Furthermore, inspired by the complex stimulus-responsive morphing that these materials exhibit, we implement hybrid particle finite element elastodynamics simulations^13^,^14^ to design non-uniform nematic director configurations that will encode a well-controlled set of folds for an auto-origami box actuator. Two nematic configurations are proposed for implementation on a cross-shaped thin film. The first proposed director microstructure is based on domains with 90-degree twist between top and bottom substrates. Twist domains are locally patterned outside the borders of the cross’s inner square. Otherwise, director is uniform along the sample thickness. The second proposed director microstructure is based on the four-fold actuation exhibited on thin films imprinted with a +1 azimuthal disclination on one substrate and a +1 radial disclination on the other substrate, a hybrid “radial-azimuthal” director configuration proposed and studied by Modes *et al*.^15^. A circular “radial-azimuthal” domain aligned with the sample center, will induce out-of-plane folds in the cross sample also creating a box morphology. This results illustrate how FEM simulations can further study complex shape evolution of samples based on well-controlled nematic microstructures and engineer stimuli-responsive polymeric devices^8^,^9^,^16^,^17^.

## Methods

For finite element simulation, an LCE thin film is discretized into a three-dimensional unstructured tetrahedral mesh using an open source software for pre- and post-processing of numerical simulations^18^. The non-uniform imprinted microstructure of the nematic director field is treated as a piecewise constant function defined in the body coordinate system. The simulation follows a Hamiltonian^13^


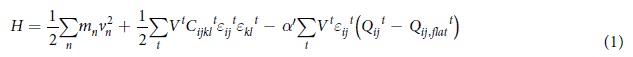


where the system’s kinetic energy is calculated using the nodes of the mesh. Node mass *m*_*n*_ is given by a lumped mass approximation on adjunct tetrahedral elements^19^, and *v*_*n*_ is the instantaneous velocity of each node. Elastic energy matches an isotropic elastic solid and it is calculated as a sum over all tetrahedral elements. The Green-Lagrange nonlinear strain tensor^3^ is given by 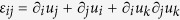. Strain tensor is coupled with the elastic stiffness tensor *C*_*ijkl*_ calculated for an isotropic elastic solid, following the relation *C*_*xxxx*_ = *C*_*xxyy*_ + 2*C*_*xyxy*_, where *C*_*xyxy*_ = *μ* and *C*_*xxyy*_ = *λ* are the Lamé coefficients^20^. An additional term corresponds to the nematic energy of a liquid crystal elastomer with an *α*′ coupling of the non-linear strain tensor with the nematic order parameter tensor, *Q*_*ij*_ = *S(n*_*i*_*n*_*j*_ − *δ*_*ij*_/3). *S* is a scalar characterizing the degree of order in the liquid crystalline phase, and 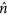 the nematic director describing the average orientation of the mesogens at the time of crosslinking. The coupling constant *α’* arises from an optimized physical crosslinking of the mesogens and polymer matrix. Note that a high-crosslinked density affects the director stimulus-response properties as well as polymer elastic constants, more details can be found on previous experimental work^21^,^22^. In this work we assume that, while the polymer matrix elastic properties are still rubber-like, *the nematic director field* is strongly cross-linked with the polymer network^23^ and as a result 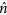 is not distorted by inner strains. Thus, changes in the nematic order parameter tensor only appear as a variation of the scalar order parameter *δS*.

The force driving node displacement is calculated as the derivative of the potential energy with respect to position. Equations of motion are integrated numerically using the velocity-Verlet algorithm. We use a dissipative force proportional to the node momentum for damping local inner-strains oscillations while allowing the system to reach equilibrium, an approach appropriate for the current system of study. As common in finite element methods, accurate results depend on a careful fine-mesh discretization along with a well-matched simulation time step. E.g. Simulations with a too-coarse mesh and/or a too-large time step can produce tetrahedral elements with negative volume (turn them inside-out), giving non-physical results. A detailed description of the hybrid-particle finite element elastodynamics algorithm can also be found in^2^,^14^,^24^.

To study the shape selection as a function of width/thickness, we generate a set of ten sample geometries with aspect ratio (*l-w*-*t*) varying from 500-25-10 to 500-250-10. Director has a smooth 90° twist along sample thickness. During the initial 125 000 time steps, *S* smoothly decreases until it reaches complete isotropy. Temperature is given by *T/T*_*NI*_ = 1.01 − (*αS*/3.03)^3/2^, where *α* = *α*′/*C*_*xyxy*_ - see Supplementary information for details on *S(T)*. In our studies of macroscopic chirality reversal in TNE, *S* smoothly changes until reaching the desired value. In both cases, after the initial variation of *S*, the system is allowed to reach equilibrium without further changes in the nematic order. The pitch and diameter of the chiral structures are determined by fitting a helix to the node positions in an edge of the ribbon^25^.

To design and engineer an auto-origami box actuator based on complex director microstructures, we generate samples with a crossed morphology. Outer cross can be enclosed within a square of aspect ratio 60-60-1. Cross maximum length, *b = 3a*; minimum length *a* = 200 and thickness *t* = 10, mesh has 55343 nodes and 295548 tetrahedral elements. The simulation on square sample with hybrid microstructure of +1 nematic disclinations was performed with a mesh of aspect ratio 50-50-1, 52780 nodes and 268246 tetrahedra. Changes in nematic order are applied according to the aforementioned protocol.

### Twist-nematic microstructure and macroscopic chiral morphology

**Figure 1:**
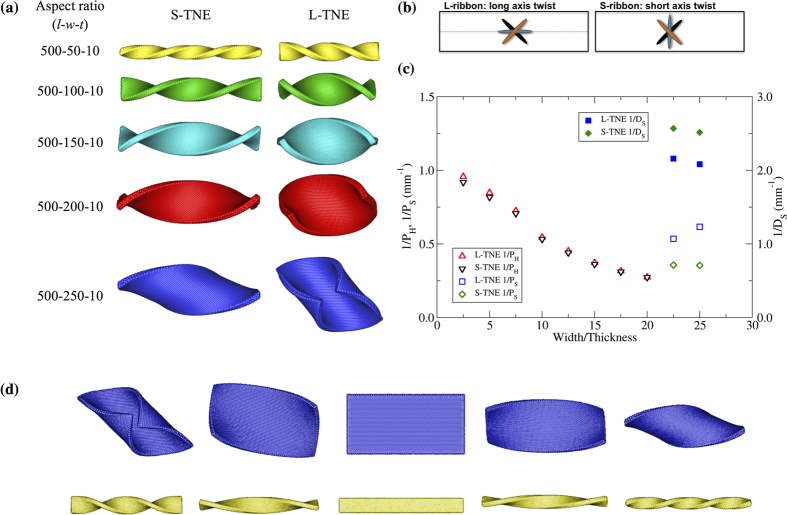
Twist-nematic elastomer ribbons: (**a**) Equilibrium state after 3 000 000 time steps of finite element simulation. S- and L- twisted nematic elastomer with different aspect ratio. Samples are heated to the high-*T* range (*T* > *T*_*flat*_), *T/T*_*NI*_ = 1.01 (*αδS* = −2.57). Elastomers with *w/t* ≤ 20 twist into helicoid ribbons and samples above this threshold width/thickness form spirals. (**b**) Schematics of 90° TNE L- and S-ribbons: Nematic orientation changes smoothly by 90° from bottom to top surface. (**c**) FEM width/thickness effect on the shape selection of TNE. Triangles represent the inverse pitch of the helicoids (Red: L-geometry; Black: S-geometry). Squares and diamonds describe the spiral ribbons (Square: L-geometry; Diamond: S-geometry), open data points represent inverse spiral pitch and filled data points represent inverse diameter. (**d**) Equilibrium state of S-TNE sample driven by different changes in nematic order, from left to right: *αδS* = 2.57, 1.28, 0.0, −1.28, −2.57. Wide sample (500-250-10) distorts into a helicoid ribbon with small changes in order parameter while large variations of *S* induce spiral ribbons. Narrow elastomer (500-50-10) always forms helicoid ribbons without dependence on *αδS*. In both samples we observe chirality reversal at the macroscopic level: *αδS* < 0 produce left-handed ribbons and *αδS* > 0 form analogous shapes with opposite chirality.

Finite element simulations of nematic elastomers with on-axis chiral director microstructure (S- and L-geometries) show the rise of chiral ribbons with temperature – Fig. 1. In the isotropic state, S-elastomers form left-handed structures – helicoid and spiral – while L-elastomers form right-handed ribbons. Other numerical studies on chiral self-assembly by Selinger, *et al*.^26^, have reported the formation of spiral ribbons with opposite chirality depending on the orientation of uniform molecular tilt related to the short and long axis of the ribbon. They showed how a 90° rotation of the tilt direction reverses the handedness of the self-assembled chiral ribbon, and greatly changes its pitch and radius. We observe an analogous situation with the nematic director in the sample mid-plane: orientation along either the short or long axis produces opposite macro-chirality.

Our simulations well-demonstrate the shape selection between helicoid and spiral ribbons depending on sample aspect ratio in both S- and L-geometries. Samples with small width/thickness (*w/t ≤ *20) form helicoid ribbons with changes in nematic order. Eventually a threshold width/thickness is reached (20 < *w*_*c*_*/t < *22.5) and spiral ribbons become the equilibrium state of the system, helicoid ribbons remain as an unstable state. Samples with aspect ratio 500-225-10 form spiral ribbons for both S- and L-geometries when the material reaches the isotropic state. The pitch of helicoid ribbons obtained with the two geometries is equivalent, and it increases as the width-to-thickness ratio increases. However, after the shape transition occurs, the spiral pitch of the S-TNE is smaller than the L-spiral pitch, which agrees with previous studies of TNE shape evolution^10^.

Comparing the resulting chiral structures observed in S- and L-elastomers, there is a notable increment of the S-TNE ribbon length. In the S-geometry, the average nematic director is perpendicular to the sample long axis; as a consequence the sample length increases in the high temperature range (*T > T*_*flat*_). In the L-geometry case, the average director orientation is parallel to the sample long axis producing a contraction of the sample length when *T* increases. The shape evolution of TNE strips above the threshold width/thickness gives interesting details about the stability of spirals and helicoids in the system. The wide TNE sample initially twists into a helicoid ribbon; after some transient time, we observe a symmetry breaking and the formation of a spiral ribbon. The time-evolution of the system’s potential energy suggests the helicoid ribbon remains as an unstable equilibrium state in the shape evolution of wide S- and L-TNE, even though the sample aspect ratio is above the threshold value for shape selection.

Selection of macroscopic chirality in TNE ribbons depending on temperature was also observed by modeling the shape deformation of two elastomers with S-geometry: one sample below the threshold width/thickness (aspect ratio 500-50-10) and another sample above the threshold (500-250-10). Even though their microscopic chirality is fixed as right-handed, both S-elastomers show reversal of its macroscopic chirality with temperature: chiral ribbons in the high-*T* range are left-handed, opposite handedness is present in the low-*T* range – Fig. 1d.

The wide elastomer shows a noteworthy shape selection with temperature: small variations of the nematic order parameter, *δS,* produces helicoids, even though its aspect ratio is above the threshold value for shape selection. At the same time, large variations of *S* induce the wide elastomer to form spiral ribbons. The observed shape selection depending on the magnitude of *δS* confirms previous analytical predictions of shape sequences in the system^10^ and agrees with the hypothesis that helicoid ribbons remain as an unstable equilibrium state in wide TNE. Experimental studies were unable to observe such phenomena because of the high sensitivity of TNE samples to small thermal variations. Our numerical study clearly shows that wide TNE can form helicoid ribbons when *T* is finely tuned and controlled. In the case of narrow TNE-S ribbon, the equilibrium state is always a helicoid with no dependence on the magnitude of nematic order change. Our results for narrow ribbons agree with both analytical studies and experimental observations.

### Auto-origami box actuator

**Figure 2:**
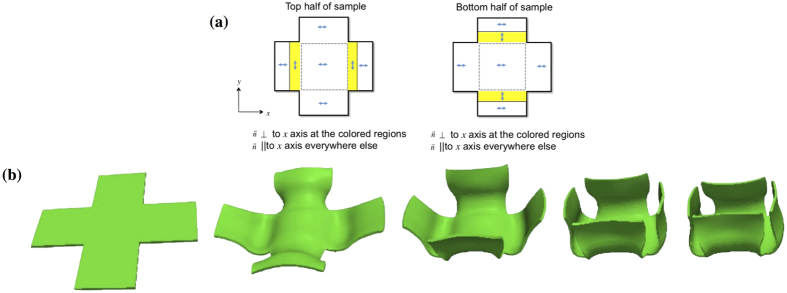
Twist-nematic domains encoding folds: Director field ***n*** for (**a**) top and (**b**) bottom substrates: ***n*** perpendicular to *x*-axis within the colour regions; ***n*** is parallel to the *x*-axis otherwise. (**c**) Crossed – Self-folding box actuator. Time-evolution of the actuation (*αδS* = +2.28) programmed by the imprinted director field. Mismatch between the director in the top and bottom substrates drives non-uniform internal strains and produces folds on a set area inside outer-squares.

Given the accuracy of our model for shape and chirality distortions in TNE ribbons, we designed a set of director microstructures that will encode a specific set of folds under external-stimulus and form a box. The self-folding-box actuator is based on a thin-elastomer film with a crossed form. Recent work by Fuchi, *et. al*. investigated a box actuator driven by optimized localization of hinges extending inside the inner square of the cross. The twist-nematic microstructure presented here localizes mismatched anchoring domains with width *w* = *a*/2, outside the cross inner square, which stabilizes its folding actuation. Figure 2a shows schematics on the director configuration within the cross sample geometry, outer square aspect ratio 60-60-1, inner square aspect ratio 20-20-1. Under external stimulus with significant variations in orientational order, *αδS < *−1.43 or *αδS* > 2.28, non-uniform strain localized on the edges of cross inner-square drives four well-controlled folds in the sample. For smaller changes in nematic order, −1.43 < *αδS* < 2.28, the box-folding actuation is distorted due to the interaction between non-uniform strains in adjunct domains within the sample. Figure 2b (Supplementary video) portraits the morphing transitions of this actuator in the low temperature range (*T* < *T*_*flat*_). Outer border of the sample shows a necked-profile corresponding with the mismatched domains highlighted in Fig. 2a.

**Figure 3:**
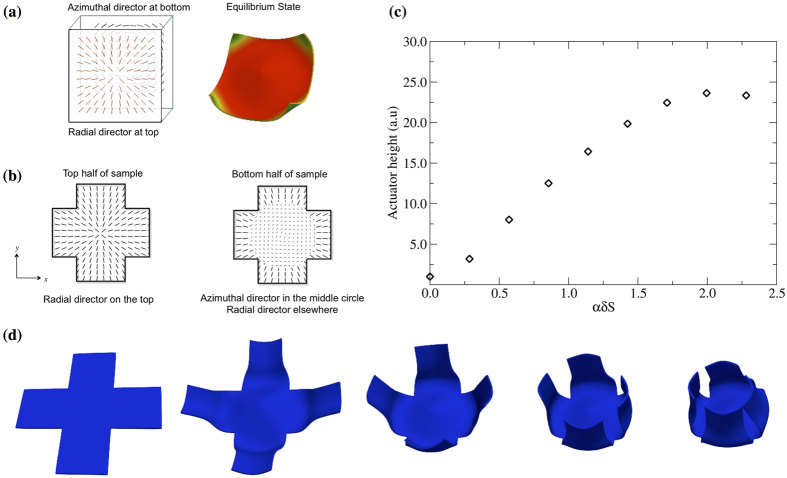
Disclination defects and out-of-plane actuation: (**a**) Director configuration and stimulus-response morphology of the radial-azimuthal membrane: +1 azimuthal disclination on the bottom half and +1 radial defect on the top half give raise to complex shape distortion when heated above crosslinking temperature. FEM simulations - sample aspect ratio is 50-50-1. (**b**) Director configuration on the top and bottom surfaces of the self-folding box film. (**c**) Actuator Height*, h = *|*z*_*max*_ − *z*_*min*_|, measured in the final state of the simulation as a function of *αδS* > 0. (**d**) Time evolution of simulated actuation in the low-temperature range (*αδS* = + 2.28), below crosslinking temperature – outer square aspect ratio 60-60-1, inner square aspect ratio 20-20-1.

The well-known relation between disclination defects, topology and curvature^27^,^28^,^29^ can also be implemented in designing and engineering a folding box actuator. A hybrid sample with radial director configuration on the top substrate aligned with an azimuthal director configuration in the bottom substrate, known as the “radial-azimuthal” actuator, distorts its shape via four-fold symmetry bending under external stimulus^15^. Figure 3a shows nematic director configuration in this actuator in a square sample and its deformation in the high*-T* range (*αδS* = −2.28). The four-fold symmetry distortion is direct consequence of the non-uniform director field. Our simulation highlights regions with high-local-internal strains. These results show agreement with theoretical predictions and experimental observations in similar systems with radial-azimuthal hybrid alignment^8^. Given these findings, we also tested the radial-azimuthal director microstructure as a suitable candidate in the design of a self-folding box. For this, the “radial-azimuthal” director pattern is imprinted within a circular region with radius *R* = 5*a*/6 centered in the thin-film. Outside this circular region, director configuration is radial and uniform along the sample thickness. Fig. 3b–d (Supplementary video) shows details of the complex director field configuration based on “radial-azimuthal” disclinations and the corresponding shape deformation in the low-temperature range (*αδS *≤ 2.28). Contrasting the twist-domain box actuator, simulations of the radial-azimuthal box show well-controlled folding actuation for small changes in nematic order. However, the programmed shape in the equivalent high-temperature range (*αδS = −*2.28) does not fully correspond with a box given the emergence of a cone-like deformation in the sample’s central region. Samples in Figs 2 and 3 are modelled with a discontinuous director in the *z*-axis. Previous numerical studies show that a *z*-continuous director twist – with defined chirality – has a small effect in the macroscopic handedness of the folds^24^, thus we can expect a similar stimulus-response.

The complex director patterns proposed in this work can be achieved using current photolithography substrate treatment techniques for inducing non-uniform liquid crystal directors^2^ and alignment on top and bottom surfaces. Our finite element simulation studies not only have successfully reproduced complex shape and chirality transformations in agreement with experimental studies and analytical theory. These simulations studies allow to design and test complex liquid crystal director microstructures for engineering folding macrostructures in thin-polymeric films. A self-folding box can be achieved by implementing the director structures proposed here.

## Conclusions

Stimulus-responsive macroscopic shape transitions can be encoded in thin-film polymeric materials by imprinting non-uniform liquid crystal director fields in an otherwise isotropic network. Here we have demonstrated a variety of out-of-plane morphing transformations including twist, chiral bending and four-fold frustration-driven bending. Such imprinted actuations depend on director geometry, sample aspect ratio as well as temperature. Even though shape selection in on-axis TNE ribbons depends strongly on sample width-to-thickness ratio, we observe that the reversal of macroscopic chirality in these materials as a function of temperature is independent of sample aspect ratio. Via FEM studies we have demonstrated this phenomena in agreement with previous experimental and analytical studies of twist-nematic elastomers. Also we have shown that helicoid/spiral shape selection in thin-film ribbons with on-axis twist nematic director does not only depend on width/thickness ratio but also on magnitude of temperature change.

Via numerical simulations we also design an auto-origami box actuator whose microstructure encodes a set of predetermined folds and out-of-plane distortions. Such macroscopic morphology can be achieved via patterning well-defined regions with a 90-degree twist in nematic director orientation between a top and bottoms surfaces of the thin film. These folds can also be achieved by implementing the four-fold symmetry out-of-plane distortion observed in thin liquid crystal elastomer films that combine radial and azimuthal disclinations with +1 topological charge, the “radial-azimuthal” configuration. Director microstructures proposed in this work provide insight in the design of stimulus-responsive materials as actuators, sensors and artificial muscles.

In summary, finite element studies provide a link between complex macroscopic qualities in liquid crystal polymeric films as chirality and shape transitions under external stimulus and the material orientational microstructure. The hybrid particle finite element elastodynamics method proves to be a strong machinery to study three-dimensional deformations in liquid crystal elastomers imprinted with non-uniform nematic director.

## Additional Information

**How to cite this article:** Gimenez-Pinto, V. *et al*. Modeling out-of-plane actuation in thin-film nematic polymer networks: From chiral ribbons to auto-origami boxes via twist and topology. *Sci. Rep.* **7**, 45370; doi: 10.1038/srep45370 (2017).

**Publisher's note:** Springer Nature remains neutral with regard to jurisdictional claims in published maps and institutional affiliations.

## Supplementary Material

Supplementary Information

Supplementary Video 1

Supplementary Video 2
