# Supplementary material for: Modeling out-of-plane actuation in thin-film nematic polymer networks: From chiral ribbons to auto-origami boxes via twist and topology
Source: Sci Rep. 2017 Mar 28;7:45370. doi: 10.1038/srep45370 (PMC5368972; doi:10.1038/srep45370)
Supplement: Supplementary Information [file srep45370-s1.pdf]

Supplementary Information

Modeling out-of-plane actuation in thin-film nematic polymer networks: From chiral ribbons to auto-origami boxes via twist and topology

Vianney Gimenez-Pinto, [a,b,c] Fangfu Ye, *[a] Badel Mbanga,[b,d] Jonathan V. Selinger,[b] and Robin L. B. Selinger*[b]

[a] *Beijing National Laboratory for Condensed Matter Physics and CAS Key Laboratory of Soft Matter Physics, Institute of Physics, Chinese Academy of Sciences, Beijing 100190, China*

[b] *Kent State University, Liquid Crystal Institute, Kent OH, United States.*

[c] *Department of Chemical Engineering, Columbia University, New York, NY, United States*

[d] *Chemical and Petroleum Engineering Department, University of Pittsburgh, Pittsburgh, PA United States*

* *Corresponding authors: fye@iphy.ac.cn and rselinge@kent.edu*

Model validation, temperature change and nematic order parameter

Finite element simulation mimics external stimulus by adjusting the order parameter *S*. In the case of thermal-responsive materials, *T* and *S* are related by $T/T_{NI} = 1.01 - \left(\alpha S/3.03\right)^{3/2}$, where α is the ratio between α' and the material shear modulus, $\mu = C_{xyxy}$. This form was obtained by fitting experimental and analytical data of the elongation Λ of a nematic elastomer with director uniformly oriented along the sample length[1,2]. We performed an analogous simulation of this system and measured the relative elongation of a nematic elastomer as a function of *S*. Figure S1a compares our simulation results with analytical predictions for Λ, which validates the relation between *T* and *S* in our simulation studies. Figure S1b shows elongation Λ as a function of temperature in our simulation vs. analytical theory.

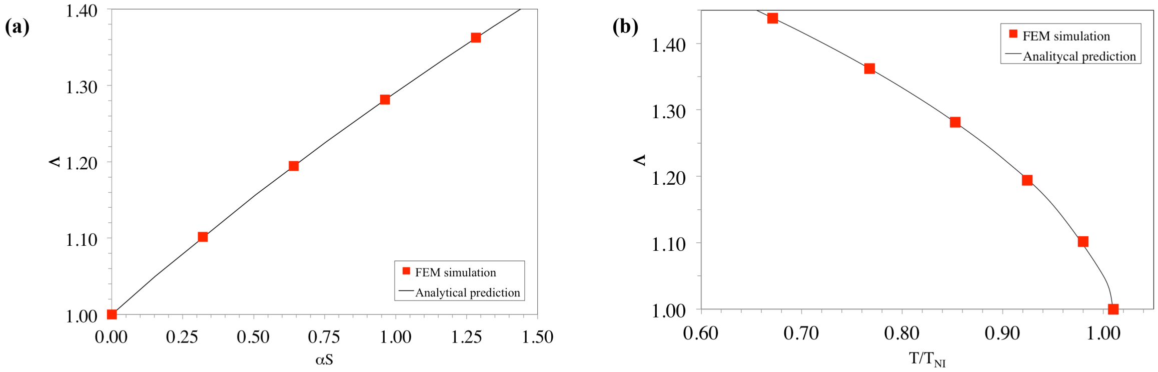

Fig. S1. (a) Elongation of elastomer with uniform nematic director vs. changes in nematic order: red square points correspond with FEM simulation; black line is analytical prediction. (b) Elongation as a function of temperature given by our simulation and analytical theory.

Finite element elastodynamics simulations

Video S1 (online)
Auto-origami box actuator: Patterned twist-nematic microstructure.
Out-of-plane actuation in the low-T range with $\alpha\delta S = 2.28$.

Video S2 (online)
Auto-origami box actuator: Centered radial-azimuthal microstructure.
Out-of-plane actuation in the low-T range with $\alpha\delta S = 2.28$.

References

1. Sawa, Y. *et al.* Shape selection of twist-nematic-elastomer ribbons. *Proc. Natl. Acad. Sci. U. S. A.* **108,** 6364–8 (2011).
2. Sawa, Y. *et al.* Shape and chirality transitions in off-axis twist nematic elastomer ribbons. *Phys. Rev. E* **88,** 22502 (2013).
